# Supplementary material for: Instability of the NS1 Glycoprotein from La Reunion 2018 Dengue 2 Virus (Cosmopolitan-1 Genotype) in Huh7 Cells Is Due to Lysine Residues on Positions 272 and 324
Source: Int J Mol Sci. 2021 Feb 16;22(4):1951. doi: 10.3390/ijms22041951 (PMC7920422; doi:10.3390/ijms22041951)
Supplement: Supplementary file 1 [file ijms-22-01951-s001.pdf]

International Journal of *Molecular Sciences*

MDPI

Instability of the NS1 Glycoprotein from La Reunion 2018 Dengue 2 Virus (Cosmopolitan-1 Genotype) in Huh7 Cells Is Due to Lysine Residues on Positions 272 and 324

Supplementary materials:

```
1                                                                             80
DSGCVVSWKNKELKCGSGIFITDNVHTWTEQYKFQPESPSKLASAIQKAHEEGICGIRSVTRLENLMWKQITPELNHILS RUJul
-------------------------------------------------------------------------------- D2_K2_RIJ_059
--------R----------------------------------------------------------------------- DENV-2/PK/2010
-------------------------------------------------------------------------------- GZ40

81                                                                           160
ENEIKLTIMTGDIKGIMQAGKRSLRPQPTELKYSWKTWGKAKMLSTEPHNQTFLIDGPETAECPNTNRAWNSLEVEDYGF RUJul
---V-------------------------------------------L--H----------------------------- D2_K2_RIJ_059
---V--S------------------------------------------------------------------------- DENV-2/PK/2010
---V-------------------------------------------L--H----------------------------- GZ40

161                                                                          240
GVFTTNIWLKLKERQDVFCDSKLMSAAIKDNRAVHADMGYWIESALNDTWKIEKASFIEVKSCHWPKSHTLWSNGVLESE RUJul
-------------------------------------------------------------------------------- D2_K2_RIJ_059
-------------------------------------------------------------------------------- DENV-2/PK/2010
-------------------------------------------------------------------------------- GZ40

241                                                                          320
MIIPKNFAGPVSQHNYRPGYHTQTAGPWHLGKLEMDFDFCEGTTVVVTEDCGNRGPSLRTTTASGKLITEWCCRSCTLPP RUJul
-------------------------------R------------------------------------------------ R D2_K2_RIJ_059
-------------------------------------------------------------------------------- DENV-2/PK/2010
-------------------------------R------------------------------------------------ GZ40

321                            352
LRYKGEDGCWYGMEIRPLKEKEENLVNSLVTA RUJul
---R---------------------------- D2_K2_RIJ_059
---R---------------------------- DENV-2/PK/2010
---R---------------------------- GZ40
```

Figure S1. Alignment of DENV-2 NS1 sequences. Alignment of DENV-2 NS1 sequences from RUJul (Reunion Island 2018), D2_K2_RIJ_059 (Tanzania 2014), DENV-2/PK/2010 (Pakistan 2010) and GZ40 (China 2010).

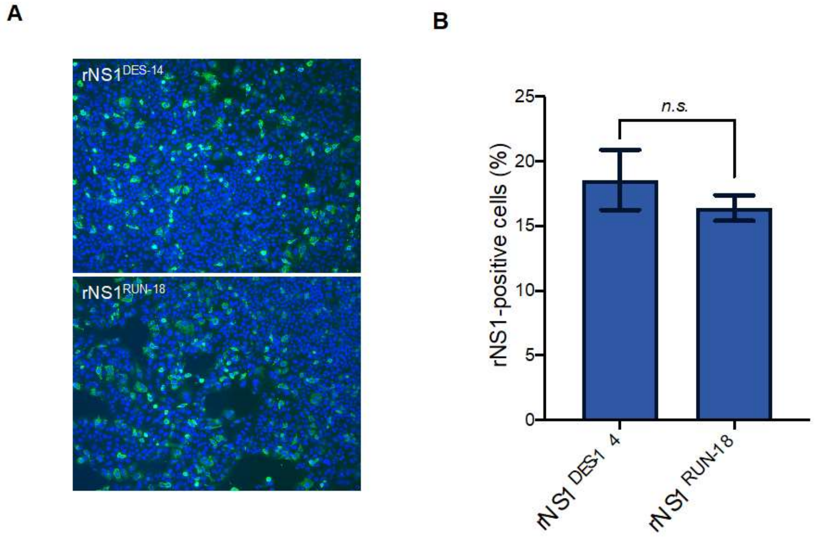

Figure S2. Expression of recombinant DENV-2 NS1 proteins in Huh7 cells. Huh7 cells were transfected 24h with pcDNA3 plasmids expressing rNS1^{DES-14} or rNS1^{RUN-18} (2.5 µg DNA per 10^6 cells). Immunofluorescence assays were performed using anti-6x(His) anti-

body (green) as primary antibody. The nuclei were stained with DAPI (blue). In (**A**), immunostained cells were visualized by fluorescence microscopy. The same magnification of x100 was used throughout. In (**B**), percentages of transfected cells immunostained with anti-6x(His) antibody. The results are the mean (± SEM) of six replicates (*n.s.*: not significant)

Table S1. Characteristics of DENV-2 clinical isolates in Indian ocean and South East Asia.

DENV-2 strain	DENV-2 genotype	Year of isolation	Place of isolation	Genbank accession number	Reference
RUJul[a]	Cosmopolitan	2018	La Reunion	MN27404	1
D2_K2_RIJ_059[b]	Cosmopolitan	2014	Tanzania[c]	MG189962	12
DENV-2/PK/2010	Cosmopolitan	2010	Pakistan	KF360005	40
GZ40	Cosmopolitan	2010	China	JX470186	41

(a). Also intitled DENV-2 strain RUN-18

(b). Also intitled DENV-2 strain DES-14.

(c). Dar es Salaam.
